# Pathoanatomy and Injury Mechanism of Typical Maisonneuve Fracture

**DOI:** 10.1111/os.12733

**Published:** 2020-09-07

**Authors:** Jin‐quan He, Xin‐long Ma, Jing‐yi Xin, Hong‐bin Cao, Nan Li, Zhen‐hui Sun, Gui‐xin Wang, Xin Fu, Bin Zhao, Fang‐ke Hu

**Affiliations:** ^1^ The First Department of Foot and Ankle Surgery Tianjin Hospital Tianjin China

**Keywords:** Ligament, Maisonneuve fracture, Medial malleolus, Posterior malleolus

## Abstract

**Objective:**

Maisonneuve fracture is a special type of injury which are rare in clinic. The manifestation of such fractures is variable. The aim of this study is to describe the pathoanatomical features of typical Maisonneuve fracture on the basis of radiographs, computed tomography (CT) scans, magnetic resonance imaging (MRI), and intraoperative exploration findings, and to investigate the injury mechanism of this variety.

**Methods:**

The data of 41 patients with Maisonneuve fracture from April 2014 to September 2019 were retrospectively analyzed. There were 32 males and nine females, the average age was 37.9 years (range, 18 to 61 years), the fractures occurred on the left side in 20 patients and on the right side in 21 patients. The cause of injuries were traffic accident in five patients, sprain injury in 20 patients, and falling injury from height in 16 patients. All patients underwent posteroanterior and lateral X‐ray examinations of the ankle and calf. CT scan of the ankle was performed in 38 patients, including three‐dimensional reconstruction in 33 patients. MRI examination of the ankle and calf was performed in 28 and five patients, respectively. Forty patients were treated with open reduction and internal fixation. The features of proximal fibular fracture, injuries of the medial and posterior structures of the ankle, injuries of the anterior inferior tibiofibular ligament and the interosseous membrane were recorded and analyzed.

**Results:**

Forty‐one patients had proximal one‐third fractures of the fibula including six patients with fracture involving the fibular neck, 30 with proximal one‐third fractures of the fibular shaft, and five with proximal–medial one‐third junction fracture of the fibular shaft. Thirty‐five patients (35/41, 85.37%) with injury of posterior structures, 34 patients had posterior malleolar fracture (34/41, 82.93%), and one patient had posterior inferior tibiofibular ligament rupture (1/41, 2.44%). There were 20 patients with type I fracture, four patients with type II fracture, and 10 patients with type III fracture according to the Haraguchi classification of posterior malleolus fracture. The fracture of the medial malleolus was in 30 patients (30/41, 73.17%), rupture of the deltoid ligament was in 10 patients (10/41, 24.39%), and medial structures intact were in one patient (1/41, 2.44%). All 41 patients had injury of the anterior inferior tibiofibular ligament.

**Conclusions:**

Maisonneuve fracture is characterized by fractures of the proximal fibula and the complete rupture of the anterior inferior tibiofibular ligament. Pronation–external rotation is the main injury mechanism. The manifestations of typical Maisonneuve fracture including that the fibular fracture located in proximal one‐third diaphysis and the fracture line was from anterosuperior to posteroinferior.

## Introduction

Maisonneuve fracture is a special type of ankle injury. A proximal fibular fracture associated with rupture of the tibiofibular syndesmosis and the anterior fibers of the deltoid ligament caused by external rotation mechanism was described for the first time in 1840 on the basis of experiments by the French surgeon Jules Germain Francois Maisonneuve^1^, which are rare in clinic, accounting for about 5% of the ankle fractures treated by surgery^2^.

Most authors conclude that the injury mechanism of Maisonneuve fracture is pronation–external rotation injury^3^, ^4^, belonging to stage III or IV pronation–external rotation mechanism according to Lauge–Hansen classification^5^. Once injured, the medial structure is first affected, including the fracture of the medial malleolus or the rupture of the deltoid ligament, followed by the rupture of the anterior inferior tibiofibular ligament (AITFL) or avulsion fracture of the attachment point, the rupture of the interosseous ligament (IOL), the rupture of the interosseous membrane (IOM), and the fracture of proximal fibula in turn. If the violence persists, it may lead to the avulsion fracture of the posterior tibial tubercle or the rupture of the posterior inferior tibiofibular ligament (PITFL).

However, the imaging findings of Maisonneuve fracture were not constantly in clinic, although many authors^6^, ^7^, ^8^, ^9^, ^10^ mostly identified proximal fibular fracture, injury of the tibiofibular syndesmosis, and medial structure as the essential characteristics of Maisonneuve fracture, whether this kind of fracture is definitely associated with medial structural injuries is still controversial.

Previous studies reported that the medial structure remained intact without rupture of the deltoid ligament or fracture of the medial malleolus in the case of Maisonneuve fracture, suggesting that supination–external rotation could also lead to Maisonneuve fracture^11^, ^12^, ^13^. For example, Charopoulos *et al*.^13^ reported a case of atypical Maisonneuve fracture, which was confirmed by X‐ray and magnetic resonance imaging (MRI) examinations as: fracture of the fibular neck; fracture of the posterior malleolus; and partial rupture of the AITFL and anterior talofibular ligament, without rupture of the deltoid ligament and fracture of the medial malleolus. The author suggested that it might be caused by ankle plantarflexion combined with external rotation, or supination–external rotation with a small degree of plantar flexion. In addition, Bissuel *et al*.^3^ reported another variant of Maisonneuve fracture characterized by rupture of the deltoid ligament and AITFL with dislocation of the proximal tibiofibular joint, without the proximal fibular fracture. Pankovich^11^ included cases without proximal fibular fracture in the study on Maisonneuve fracture; moreover, he reported two cases of supination–external rotation high fibular fracture, the fracture line extended from the anteroinferior edge in a posterosuperior direction, which was different from the classic rendering.

At present, there is no consensus on how to describe the definition of Maisonneuve fracture. Different clinical manifestations indicate that the injury mechanism and pathological changes related to Maisonneuve fracture need further investigation and analysis. The characteristics of Maisonneuve fracture, such as the position, morphology, and extent of the injury of every component, are the main basis for inferring injury mechanism. Due to the lower morbidity of Maisonneuve fracture, very few studies with more than 20 cases are available. Also, not all studies have a detailed description of the specific injury characteristics of such fractures, bringing about difficulties in the research work to some extent.

In this study, the imaging data of 41 patients with Maisonneuve fracture in our department, including the manifestations of X‐ray, computed tomography (CT), MRI, and intraoperative exploration findings, were collected and sorted out systematically. The purpose was to: (i) describe the pathoanatomical feature of the constituents of Maisonneuve fracture, such as the fibula, medial structures, posterior structures, and syndesmosis; (ii) investigate the injury mechanism of Maisonneuve fracture; and (iii) summarize the essential characteristics of typical Maisonneuve fracture on the basis of our discovery.

## Materials and Methods

This study was approved by the Hospital Ethics Committee and all selected patients provided signed informed consent.

### *Inclusion and Exclusion Criteria*

Inclusion criteria: (i) an ankle injury with the proximal one‐third fracture of the fibula; (ii) the interval between injured and admitted less than 5 days; (iii) body mass index <25 kg/m^2^; (iv) physical examination revealed tenderness over the ankle and proximal fibula; (v) patients who had the data of CT and/or MRI besides the radiographs; and (vi) a retrospective study.

Exclusion criteria: (i) less than 18 years old; (ii) patients with osteoarthritis of the ankle; (iii) patients who had previous ankle injury or surgical history; and (iv) patients with concomitant or open injury.

### *Patients' Information*

The data of 41 patients with Maisonneuve fracture admitted to our department from April 2014 to September 2019 were retrospectively analyzed. There were 32 males (32/41, 78.05%) and nine females (9/41, 21.95%) with an average age of 37.9 years (range, 18 to 61 years). The fractures occurred on the left in 20 patients (20/41, 48.78%) and on the right in 21 patients (21/41, 51.22%). The cause of injuries were traffic accident in five patients, sprain injury in 20 patients, and falling injury from height in 16 patients.

### *Intervention*

All patients underwent posteroanterior and lateral X‐ray examinations of the ankle and calf. CT scan of the ankle was performed in 38 patients, including three‐dimensional reconstruction (3D CT) in 33 patients. MRI examination of the ankle and calf was performed in 28 and five patients, respectively. Further, 40 patients were treated with open reduction and internal fixation.

### *Outcome Measures*

The findings of X‐ray, CT, MRI, and intraoperative exploration were evaluated, and the following data were recorded and analyzed:

*Proximal Fibular Fracture*

The features of proximal fibular fracture were recorded according to the anatomical region: fibular neck, upper third of the fibular diaphysis, the junction of the middle and upper third of the fibula. The morphology of fracture: simple (spiral) or comminuted; the direction of fracture line, such as extended from anterosuperior to posteroinferior in the lateral radiographs, from laterosuperior to medialinferior in the posteroanterior radiographs.

*Injuries of the medial structures of the ankle*

Injuries of the medial structures were defined as medial malleolar fracture or deltoid ligament rupture. Medial malleolar fracture was assessed on the basis of posteroanterior radiographs, and classified into three types according to the direction of fracture line: transverse, oblique, and vertical. The fragment involving anterior colliculus, intercollicular groove, and posterior colliculus was evaluated by 3D CT reconstructions. Deltoid ligament rupture was identified with MRI. Ligament status was defined as follows: an intact ligament had a homogeneous low signal intensity without evidence of disruption or tissue edema. A partially disrupted ligament demonstrated a few intact fibers surrounded by areas of increased signal representing partial ligament tear with associated hemorrhage and edema. A complete tear demonstrated discontinuity of the ligamentous structure with surrounding areas of edema and hemorrhage^14^.

*Injuries of the Posterior Structures of the Ankle*

Injuries of the posterior structures were defined as posterior malleolar fracture or AITFL rupture. Posterior malleolar fracture was identified preliminarily with radiographs and the diagnosis was confirmed by CT scan or MRI. The morphology of the posterior malleolar fracture was evaluated by the image of transverse CT scan and classified according to the classification developed by Haraguchi^15^: type I (posterolateral‐oblique), type II (transverse medial‐extension) and type III (small‐shell fragment). Injury of the PITFL was determined according to MRI. Ligament status was defined as intact, partially disrupted, and complete tear^15^.

*Injuries of the Anterior Inferior Tibiofibular Ligament (AITFL)*

Injury of AITFL is characterized by rupture of the ligament or avulsion fracture. The AITFL extends from the anterior tubercle of the distal tibia (Tillaux–Chaput) to the anterior tubercle of the distal fibula (Wagstaffe). CT scan shows the avulsion fracture of the Tillaux–Chaput tubercle or Wagstaffe tubercle. Staus of the AITFL was identified according to MRI.

*Injuries of the Interosseous Membrane (IOM)*

Rupture of the IOM was identified on the basis of MRI of ankle and lower leg and described in light of the position: proximal, middle, and distal third. The extent of the IOM rupture is depicted by the distance to the articular surface of distal tibia. IOM status is defined as follows: an intact IOM is a homogeneous low signal intensity connecting the cortices of the tibia and fibula without disruption or evidence of oedema; a partial injury of IOM is a wavy but still continuous low signal intensity, usually surrounded by high signal intensity representing hemorrhage and oedema; a complete rupture of IOM was demonstrated by the disruption of the low signal intensity and was interrupted by the high signal intensity of hemorrhage and oedema^8^.

The aforementioned data were independently evaluated by three chief orthopaedic surgeons. In the case of any disagreement, the three surgeons discussed the injury until a consensus was reached.

## Results

### *Fracture of the Proximal Fibula*

**Figure 1:**
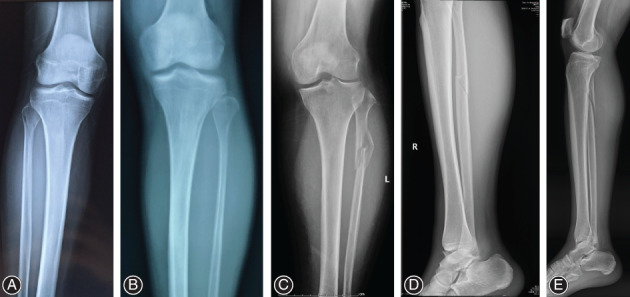
Manifestations of proximal fibular fractures. (A) Fibular neck fracture, (B) Fibular neck fracture without coronal displacement, (C) Proximal one‐third fracture of the fibula with an irregular fracture line, (D) Proximal–medial one‐third junction fracture of the fibula, (E) the fracture line extended from the anterosuperior edge in a posteroinferior direction.

The position and morphology of the fibular fracture line were evaluated using posteroanterior and lateral radiographs of the calf. It was found that 41 patients had proximal one‐third fractures of the fibula, mainly manifested as spiral or comminuted fractures, including six patients with fracture involving the fibular neck (6/41, 14.63%), 30 with proximal one‐third fibular diaphysial fractures (30/41, 73.17%), and five with proximal–medial one‐third junction fracture of the fibula (5/41, 12.20%) (Fig. 1). Posteroanterior radiographs showed that the fracture line extended from the laterosuperior edge in a medialinferior direction in 26 patients (26/41, 63.41%); no obvious fracture line and lateral radiographs were needed to confirm the fracture in nine patients (9/41, 21.95%); fracture line was irregular in six patients (6/41,14.63%). Lateral radiographs showed that the fracture line extended from the anterosuperior edge in a posteroinferior direction in 35 patients (35/41, 85.37%) and revealed comminuted fractures in six patients (6/41, 14.63%) with an irregular fracture line.(Table 1).

**TABLE 1:** Clinical characteristics of Maisonneuve fracture

	Cases	Propotion (%)
Medial structures		
Medial malleolar fracture	30	73.17
Deltoid ligament rupture	10	24.39
Intact	1	2.44
Fibular fracture	41	100
Posterior structures		
Posterior fracture	34	82.93
PITFL rupture	1	2.44
Intact	6	14.63
AITFL rupture	41	100

PITFL, posterior inferior tibiofibular ligament; AITFL, anterior inferior tibiofibular ligament.

### *Injury of Posterior Structures of the Ankle*

This study included 35 patients (35/41, 85.37%) with injury of posterior structures, posterior malleolar fracture was in 34 patients (34/41, 82.93%), and PITFL rupture was in one patient (1/41, 2.44%). Twenty‐eight posterior malleolar fractures were confirmed by radiographs of the ankle. Furthermore, CT and MRI findings ascertained the posterior malleolar fractures in 34 patients, rupture of the PITFL at the tibial insertion in one patient, and no injuries of the posterior malleolus or PITFL in six patients (in this study, three patients did not undergo CT examination but underwent MRI examination).

**Figure 2:**
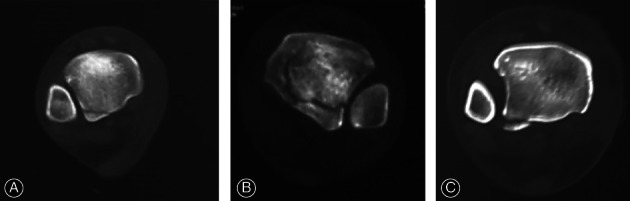
Manifestations of posterior malleolar fractures according to the Haraguchi classification. (A) Type I (posterolateral oblique) fracture, (B) Type II (transverse medial–extension type) fracture, (C) Type III (small‐shell type) fracture.

Among 13 patients without posterior structural injuries in the radiographs, posterior malleolar fracture or posterior inferior tibiofibular ligament rupture were confirmed in seven patients (7/13, 53.85%) by CT or MRI. The CT or MRI cross‐sectional images of 34 patients with posterior malleolus fracture indicated 20 patients with type I (posterolateral oblique) fracture, four patients with type II (transverse medial–extension type) fracture, and 10 patients with type III (small‐shell type) fracture according to the Haraguchi classification of posterior malleolus fracture^15^(Fig. 2).

### *Injury of Medial Structures of the Ankle*

**Figure 3:**
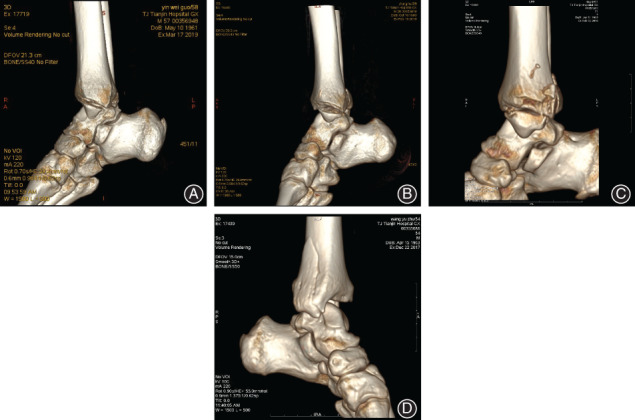
Manifestations of medial malleolar fractures. (A) Fractures of anterior colliculus and intercollicular groove (B) Fractures of anterior colliculus, intercollicular groove and a small portion of posterior colliculus, (C) Fractures of anterior colliculus, intercollicular groove and posterior colliculus, (D) Fractures of posterior colliculus, intercollicular groove and a portion of anterior colliculus.

The injury of medial structures of the ankle was comprehensively evaluated by X‐ray, CT, MRI, and intraoperative exploration. Among the 41 patients, 30 patients (30/41, 73.17%) had medial malleolus fractures. Moreover, 10 patients (10/41, 24.39%) without medial malleolus fractures were confirmed by MRI to have deltoid ligament rupture, and one (1/41, 2.44%) was confirmed by CT and MRI to have no medial malleolus fracture and deltoid ligament rupture. Based on the posteroanterior radiographs of the ankle of the 30 patients with medial malleolus fracture, 18 had transverse fractures, nine had oblique fractures, and the other three had vertical fractures. Also, 26 of the 30 patients underwent 3D CT reconstruction combined with imaging and intraoperative exploration. It was found that 13 patients had fractures involving the anterior colliculus and intercollicular groove, 11 had fractures involving the whole medial malleolus (anterior colliculus, intercollicular groove, and posterior colliculus), and two had fractures involving the posterior colliculus, intercollicular groove and a portion of anterior colliculus (Fig. 3).

### *Syndesmotic Lesions*

**Figure 4:**
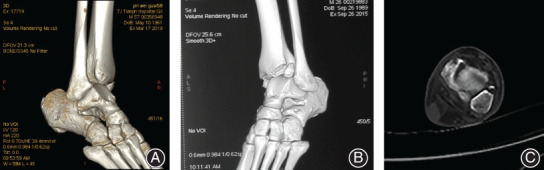
Manifestations of injury to the syndesmosis (A) Widening of the tibiofibular space with external rotation of the distal fibula, (B) Avulsion fracture of the Tillaux–Chaput tubercle, (C) Wagstaffe fracture of the distal fibula.

The injury of the inferior tibiofibular ligament was comprehensively evaluated by CT, MRI, and intraoperative exploration (direct vision, external rotation, and hook test). All 41 patients had injury of the AITFL. CT cross‐sectional images of 38 patients showed a widening of the tibiofibular space with external rotation of the distal fibula in 29, avulsion fracture of the Tillaux–Chaput tubercle in four, and Wagstaffe fracture in one (Fig. 4). Moreover, 28 patients underwent MRI examination of the ankle and all showed the rupture of the AITFL. The injury of the posterior structure was posterior malleolus fracture in 34 patients and PITFL rupture in one patient. The complete ruptures of the AITFL were found during intraoperative exploration in all 40 patients treated surgically.

### *Injury of the Interosseous Membrane*

The injuries of the distal IOM were evaluated by MRI in five patients. The complete rupture of the AITFL, the interosseous ligament and the IOM were found, with a rupture range of 6–9 cm from the articular surface of the distal tibia, with an average of 6.6 cm (Table 2). In addition, the rupture of the IOM was also present near the fibular fracture with an intact IOM between the proximal and distal ruptures in some patients.

**TABLE 2:** Rupture of the interosseous membrane

Case	Fibula	Medial structure	Posterior malleolus	AITFL	IOM (cm)
1	Superior one‐third	Complete rupture of the deltoid ligament	Fracture	Complete rupture	9
2	Superior one‐third	Fracture of the medial malleolus	Fracture	Complete rupture	6
3	Superior one‐third	Fracture of the medial malleolus	Fracture	Complete rupture	6
4	Neck of the fibula	Fracture of the medial malleolus	Fracture	Complete rupture	6
5	Superior one‐third	Fracture of the medial malleolus	Fracture	Complete rupture	6

AITFL, anterior inferior tibiofibular ligament; IOM, interosseous membrane.

### *Injury Mechanism*

Injuries of medial structures were recorded in 40 of 41 cases (97.56%), 30 patients (30/40, 75%) had medial malleolus fractures, and 10 patients (10/40, 25%) had deltoid ligament complete rupture. Ruptures of AITFL and proximal fibular fractures were identified in all 40 cases with medial structures injuries (40/40, 100%). Injuries of the posterior structures was recorded in 34 cases (34/40, 85%), including 33 fractures of the posterior malleolus (33/34, 97.06%) and one rupture of PITFL (1/34, 2.94%), posterior structures were intact in six cases (6/40, 15%). According to Lauge‐Hansen classification^5^, 40 cases with medial structure injuries were classified as pronation external rotation (PER): stage III in six cases (6/40, 15%) and stage IV in 34 cases (34/40, 85%).

One case (1/41, 2.44%) had intact medial structures, complete rupture of AITFL, proximal fibular fracture, and posterior malleolar fracture. In spite of the fact that the imaging findings were atypical, we classified this case as supination external rotation mechanism^11^, ^12^, ^13^.

## Discussion

### *Fracture of the Proximal Fibula*

Spiral fracture of the proximal one‐third fibula is one of the most important characteristics of Maisonneuve fracture; some patients can also have comminuted fractures. This study included 41 patients with fracture of the proximal one‐third fibula, six with fracture involving the fibular neck, 30 with fracture of the proximal one‐third fibular shaft, and five with fracture of the proximal–medial one‐third junction of the fibular shaft. According to Lauge–Hanson classification, fibular fracture of pronation–external rotation injuries is usually located 1 to 3 inches above the tibiofibular syndesmosis. However, it can occur anywhere from the fibular neck to the distal tibiofibular syndesmosis, with 73% located in the range of 2.5–8 cm proximal to the ankle and 10% in the area of the fibular neck^16^. In this study, 41 patients of fibular fractures were located proximal to the diatal tibiofibular syndesmosis, which had one of the characteristics of the pronation–external rotation‐type fracture of the ankle. However, it was not enough to determine the aforementioned patients as pronation–external rotation‐type injuries. The injury mechanism needed to be determined by combining injuries in other parts of the ankle.

The extension of the fibular fracture line is one of the bases to determine the mechanism of ankle injury. For example, the fibular fracture line caused by pronation–external rotation is usually from the anterosuperior to the posteroinferior direction, while the fibular fracture line caused by supination–external rotation is usually from the anteroinferior to the posterosuperior direction. However, the Maisonneuve fracture is caused by the external rotation of the foot relative to the tibia. During rotation, whether the foot is in the supination or pronation position, or whether the position of the foot changes during the injury, is not clearly described. Pankovich^11^ found that the removal of the AITFL and the external rotation of the foot in the neutral position led to the rupture of the interosseous ligament and similar pronation–external rotation fracture of the proximal fibula (the fracture line from the anterosuperior to the posteroinferior direction). Further, the removal of the AITFL and the interosseous ligament and the external rotation of the foot in the supinator position led to similar supination–external rotation fracture of the proximal fibula (the fracture line from the anteroinferior to the posterosuperior direction). Pankovich^11^ also found two cases of Maisonneuve fracture with a fracture line of the proximal fibula running from the anteroinferior to the posterosuperior direction and believed that the injury mechanism of Maisonneuve fracture included pronation–external rotation and supination–external rotation. In this study, the lateral radiographs showed that the typical fracture line of the fibula ran from the anterosuperior to the posteroinferior direction in 35 patients (35/41, 85.37%), without any evidence of the fracture line of the proximal fibula running from the anteroinferior to the posterosuperior direction. The posteroanterior radiographs showed that the majority fracture line of the fibula was from laterosuperior to medialinferior (26/41, 63.41%).

The proximal fibular fracture of the Maisonneuve fracture is prone to be missed at the first visit, with a missed diagnosis rate of 14.28%–44.4%^7^, ^9^, ^17^. It is mainly because of neglecting palpation and X‐ray examination of the whole fibula. Further, due to the absence of an obvious coronal displacement in the proximal fibular fractures and the absence of an obvious fracture line in the posteroanterior radiographs, the fractures should be confirmed by lateral radiographs. In this study, such cases accounted for 21.95% of all cases, suggesting that attention should be paid when reading the radiographs.

### *Injury of Posterior Structures of the Ankle*

The posterior inferior tibiofibular ligament was ruptured at the tibial insertion in one patient. The PITFL is relatively thick, starting from the posterior tubercle of the tibia and ending at the posterior tubercle of the fibula. The incidence of avulsion fracture of the posterior malleolus is greater than that of the ligament rupture caused by strong violence. In the process of external rotation of the fibula, the PITFL and the transverse ligament play the role of a posterior hinge; even if a fracture of the posterior malleolus occurs, it rarely involves more than one‐fourth of the joint surface^4^.

Among the 13 patients without posterior malleolar fractures on the radiographs, seven (7/13, 53.85%) had confirmed injuries of the posterior structures of the ankle on CT or MRI examination, including one with rupture of the PITFL and six with Haraguchi type III fractures of the posterior malleolus. These findings suggested that X‐ray had some limitations in the diagnosis of posterior malleolar fractures, and CT and MRI examinations should be performed to evaluate the fracture and ligament injury.

Moreover, 34 patients (34/41, 82.93%) had posterior malleolus fracture, classic manifestation of posterior malleolar fracture were posterolateral oblique (20/34, 58.82%) and small‐shell type (10/34, 29.41%) resulted from rotation mechanism.^15^ Four cases had Haraguchi type II fracture (transverse medial–extension type). The common characteristics in these four patients were that the fracture line extended from the fibular notch of the distal tibia to the medial side, and the fracture fragments were divided into the posterolateral part and the posteromedial part. The injury mechanism might be that the combined effect of rotation and axial force on the foot with ankle plantar flexion led to the impact of the talus on the posterior distal tibia, resulting in injuries characterized by Maisonneuve fracture and hyper plantar flexion–type ankle fracture.

### *Injury of Medial Structures of the Ankle*

According to the Lauge–Hanson classification, the medial malleolus fracture or deltoid ligament rupture occurs in pronation–external rotation injuries due to the first occurrence of the medial structure injuries. However, the medial structure injuries occur in the last stage in supination–external rotation injury, which is one of the bases to determine the injury mechanism of ankle fracture. This study included one patient with a rupture of the AITFL and fractures of the proximal fibula and posterior malleolus but without fractures of the medial malleolus and ruptures of the deltoid ligament confirmed by CT and MRI, which was an atypical supination–external rotation fracture. The remaining included 30 patients with medial malleolar fracture (30/41, 73.17%) and 10 with deltoid ligament rupture (10/41, 24.39%), which were pronation–external rotation injuries. The typical morphology of medial malleolar fracture was transverse (18/30, 60%) or oblique (9/30, 30%) pattern in the posteroanterior radiographs.

In patients with medial malleolus fractures, 26 underwent 3D CT reconstruction combined with intraoperative exploration. The fractures involved the anterior colliculus and the intercollicular groove in 13 patients, the whole medial malleolus (anterior colliculus, intercollicular groove, and posterior colliculus) in 11 patients, and the posterior colliculus, intercollicular groove and a portion of anterior colliculus in two patients, these two patients were classified as Haraguchi type II fractures. In this study, the fracture line of the medial malleolus extended vertically in three patients. Of these, two had Haraguchi type II fracture of the posterior malleolus involving the posterior colliculus, intercollicular groove and a portion of anterior colliculus, and one had vertical fracture of the medial malleolus involving the anterior colliculus, intercollicular groove and posterior colliculus, combined with ruptures of the AITFL and anterior talofibular ligament and proximal one‐third fracture of the fibula. It was speculated that the position of the ankle immediately changed from supination adduction to pronation–external rotation in the initial stage of the injury. Morris^14^ performed an MRI examination on five cases with acute Maisonneuve fracture. All patients had ruptures of the superficial deltoid ligament. Three cases were of complete rupture of the deep deltoid ligament, one of partial rupture of the deep deltoid ligament, and one of the intact deltoid ligaments. In conclusion, the injuries of medial structures in Maisonneuve fractures varied from complete injuries, such as fractures of the whole medial malleolus, complete ruptures of the deltoid ligament, complete fractures of the anterior colliculus with rupture of the deep deltoid ligament, to partial injuries, such as ruptures of the superficial deltoid ligament and fractures of the anterior colliculus.

### *Injury of the Inferior Tibiofibular Ligament*

Hermans^18^ believed that the X‐ray findings of the inferior tibiofibular overlap and space could not accurately reflect the inferior syndesmosis injury. Therefore, the findings of CT, MRI, and intraoperative exploration were mainly evaluated in this study. Ruptures of the AITFL occurred in all 41 patients, including four with the avulsion fracture of Tillaux–Chaput nodule and one with Wagstaffe fracture. This study and some other studies^8^, ^14^ found that all patients had injuries of the AITFL, confirming once again that injury of the AITFL was one of the essential characteristics of Maisonneuve fracture. AITFL plays a very important role in maintaining the stability of the ankle, mainly to prevent the external rotation and backward displacement of the fibula^19^. It is also the weakest ligament of the tibiofibular syndesmosis and the first structure injured when the fibula is in the position of external rotation, which can be manifested as ligament rupture or avulsion fracture of its attachment (such as Chaput tubercle or Wagstaffe fracture)^20^.

Previous studies^5^, ^13^, ^21^ suggested that the force of external rotation led to the rupture of the interosseous ligament and the conduction of the force along the IOM to the proximal end led to fibular fracture and rupture of the IOM to the level of the fibular fracture. Manyi *et al*.^8^ found that all 12 patients with Maisonneuve fractures had the injury of the IOM, but only limited to the distal one‐third of the calf and no more than 112 mm to the proximal ankle level. The MRI examination of the calf in five patients in this study found that the rupture range of the distal IOM was 6–9 cm to the distal articular surface of the tibia, with an average of 6.6 cm, reconfirming that the rupture range of the IOM was not from its distal stop to the level of fibular fracture.

The IOM is a fibrous structure that connects the tibia and fibula. It can prevent the fibula from moving to the lateral side but cannot limit fibula rotation and sagittal displacement. Merril^6^ believed that the application of abduction force on the distal fibula could lead to the rupture of the IOM, and the fracture of the proximal fibula was caused by the force of external rotation of the fibula blocked by the ligaments and joint capsule structures surrounding the superior tibiofibular joint. The present study also found rupture of the IOM near the fibular fracture in some patients, but the IOM between proximal and distal ruptures was intact. The specific mechanism remains to be further explored.

### *Injury Mechanism*

According to Lauge‐Hansen classification and imaging findings, the injury mechanism of Maisonneuve fracture can be divided into pronation external rotation and supination external rotation. In our study, one case (1/41, 2.44%) was caused by supination external rotation mechanism and 40 cases (40/41, 97.56%) was caused by pronation external rotation mechanism. Bartonícek *et al*.^12^ demonstrated that injury mechanism of 47 in 54 Maisonneuve fractures (87.04%) was pronation external rotation, and many other studies^6^, ^7^, ^8^, ^9^, ^10^ also reported that main injury mechanism of Maisonneuve fracture was pronation external rotation, which is in accordance with the finding of our study. Maisonneuve fracture is caused less by the mechanism of supination external rotation. Pankovich^11^ described five stages in injury mechanism of Maisonneuve fracture: (i) rupture of the anterior tibiofibular ligament or avulsion fracture of one of its bone insertions, either one being associated with rupture of the interosseous ligament; (ii) fracture of the posterior tubercle or rupture of the posterior tibiofibular ligament; (iii) rupture of the anteromedial joint capsule or avulsion fracture of one of its bone insertions; (iv) fracture of the proximal part of the fibula; and (v) rupture of the deltoid ligament or fracture of the medial malleolus. This mechanism can explain why the medial structures are left intact.

### *Conclusions*

Maisonneuve fracture is caused by the force of external rotation. Pronation–external rotation is the injury mechanism in most patients, but it can also be caused by supination–external rotation in some patients. It is characterized by fractures of the proximal fibula and the rupture of the AITFL. Hence, medial malleolar fracture, deltoid ligament rupture, posterior malleolar fracture, and other injuries are possible. The rupture range of the interosseous membrane is not from its distal stop to the level of fibular fracture.
